# Shaping the future of male reproductive health: fostering talent at the 14th Network of Young Researchers in Andrology meeting

**DOI:** 10.1242/bio.059901

**Published:** 2023-05-30

**Authors:** Daniel Marcu, Dorte Egeberg, Guillaume Richer, Manon Oud, Brendan Houston, Sven Berres, Alberto de la Iglesia

**Affiliations:** ^1^ Network for Young Researchers in Andrology (NYRA), 08021, Barcelona, Spain; ^2^ School of Biological Science, University of East Anglia, NR4 7TJ Norwich, UK; ^3^ European Sperm Bank Struenseegade 9, 2, 2200 Copenhagen, Denmark; ^4^ Biology of the Testis lab, University Medical Campus, Vrije Universiteit Brussel, 1090 Brussels, Belgium; ^5^ Department of Human Genetics, Donders Institute for Brain, Cognition and Behaviour, Radboudumc, 6525 GD Nijmegen, The Netherlands; ^6^ School of BioSciences, The University of Melbourne, Parkville, Victoria, 3010, Australia; ^7^ Centre for Reproductive Medicine and Andrology, University of Münster, 48149 Münster, Germany; ^8^ Université Paris Cité, INSERM, CNRS, Institut Cochin, F-75014 Paris, France

**Keywords:** Andrology, Infertility, Male fertility, Meeting review, Spermatogenesis

## Abstract

The 14th Network of Young Researchers in Andrology (NYRA) meeting was a 2-day conference held at the University of East Anglia in Norwich, UK, organized by the NYRA. The meeting brought together researchers and experts to discuss and exchange ideas on male infertility and spermatogenesis. The meeting covered a wide range of topics related to male germline research, including the impact of mutations in the male germline on future generations, the use of innovative sequencing tools for the study of male infertility, and the intricate germline epigenome. The impact of aging on spermatogenesis was also discussed, with a focus on the increased DNA fragmentation rates, changes in DNA methylation patterns, and longer telomeres associated with aging sperm. Additionally, progress on fertility preservation options for children undergoing gonadotoxic cancer treatments was presented. The meeting also featured workshops on leadership and career development strategies in science, providing a valuable opportunity for young researchers to learn from experts in the field and exchange ideas with their peers. Overall, the meeting provided a platform for researchers to discuss the latest developments in male germline research, highlighting the importance of empowering young researchers to tackle male reproductive health.

## Teaser

**Summary:** The 14th NYRA meeting covered a wide range of topics related to male germline research, including the impact of mutations in the male germline on future generations, the use of innovative sequencing tools for the study of male infertility, and the intricate germline epigenome.

## Introduction

The worldwide decline in fertility rates is a matter of great concern. Indeed, infertility, defined by the inability to conceive after one year of regular unprotected sexual intercourse, affects an estimated 15% of all couples (Agarwal et al., 2021; Murray et al., 2018; Skakkebaek et al., 2019). Although male factors contribute to 50% of the infertility cases (Agarwal et al., 2021; Levine et al., 2022; Salonia et al., 2021; Tiegs et al., 2018), studies of the underlying causes have been biased towards the examination of women. This may be influenced, in part, by social factors that have historically discouraged men from seeking routine health care unless in acute medical conditions (de Jonge et al., 2023).

More specifically, numerous studies have reported a declining trend in sperm quality over the past 60 years (Agarwal et al., 2021; Levine et al., 2017, 2022; Salonia et al., 2021; Tiegs et al., 2018). Poor sperm quality is associated with compromised male reproductive health and interrelated testicular dysgenesis syndrome, including testicular cancers, disturbed spermatogenesis, cryptorchidism and hypospadias, and ultimately results in an increased demand for assisted reproduction techniques (Skakkebaek et al., 2001, 2016, 2019). Male infertility has a complex etiology, which encompasses various potential causes including anatomical defects, genetic disorders, endocrine imbalances, and immune system abnormalities, among others (Salonia et al., 2023; Minhas et al., 2021; Tiegs et al., 2018). However, it is concerning that the causes of approximately half of male infertility cases remain elusive (Agarwal et al., 2021). Furthermore, sociodemographic factors, such as delayed childbearing and lifestyle can affect male reproductive health, which may be perpetuated to the offspring (Sharma et al., 2013; Tiegs et al., 2018; Balawender and Orkisz, 2020; Agarwal et al., 2021).

Despite the lack of attention given to male infertility, andrology – the medical specialty that focuses on the study of male reproductive health and its disorders – is gaining recognition, albeit slowly, in some countries (Schlegel et al., 2021). Initiatives, such as Andrology Awareness, the COST Action ANDRONET, Fertility Europe, and Healthy Male raise awareness of this field, and promote education and research into the diagnosis, prevention and treatment of male reproductive disorders. Moreover, the Network for Young Researchers in Andrology (NYRA) was established in 2006 under the name of ‘Young Testis Club’, with the aim of providing a platform for young researchers in andrology to exchange knowledge, foster new research collaborations, and develop soft skills through workshops and annual meetings (Tüttelmann et al., 2012; Amaral et al., 2012). With its focus on early-career researchers, the Network for Your Researchers in Andrology (NYRA) has become a hub for cutting-edge research and discussions on male infertility, bringing together MSc students, PhD candidates, and early postdoctoral researchers along with internationally renowned experts. By supporting the next generation of researchers in this field, NYRA is playing a vital role in advancing andrology research to offer solutions to the pressing issue of male infertility.

## The 14th NYRA meeting

The 14th NYRA meeting was initially organized to host international young researchers in andrology for a period of 3 days, from 19–21 September 2022, at the University of East Anglia (UEA) in Norwich, UK. The UEA is part of the Norwich Research Park, one of Europe's biggest communities of researchers in the areas of environment, health, and plant science. However, the unforeseen passing of Queen Elizabeth II of the UK on 8 September 2022 altered our original plans, as the state funeral coincided with the first day of our meeting and was officially designated as a national day of mourning. Nonetheless, we remained committed to maintaining the scientific content of the event by rescheduling all scientific sessions and workshops within 1.5 days, while still providing the attendees sufficient free time to explore the wonderful city of Norwich.

**Fig. 1.**
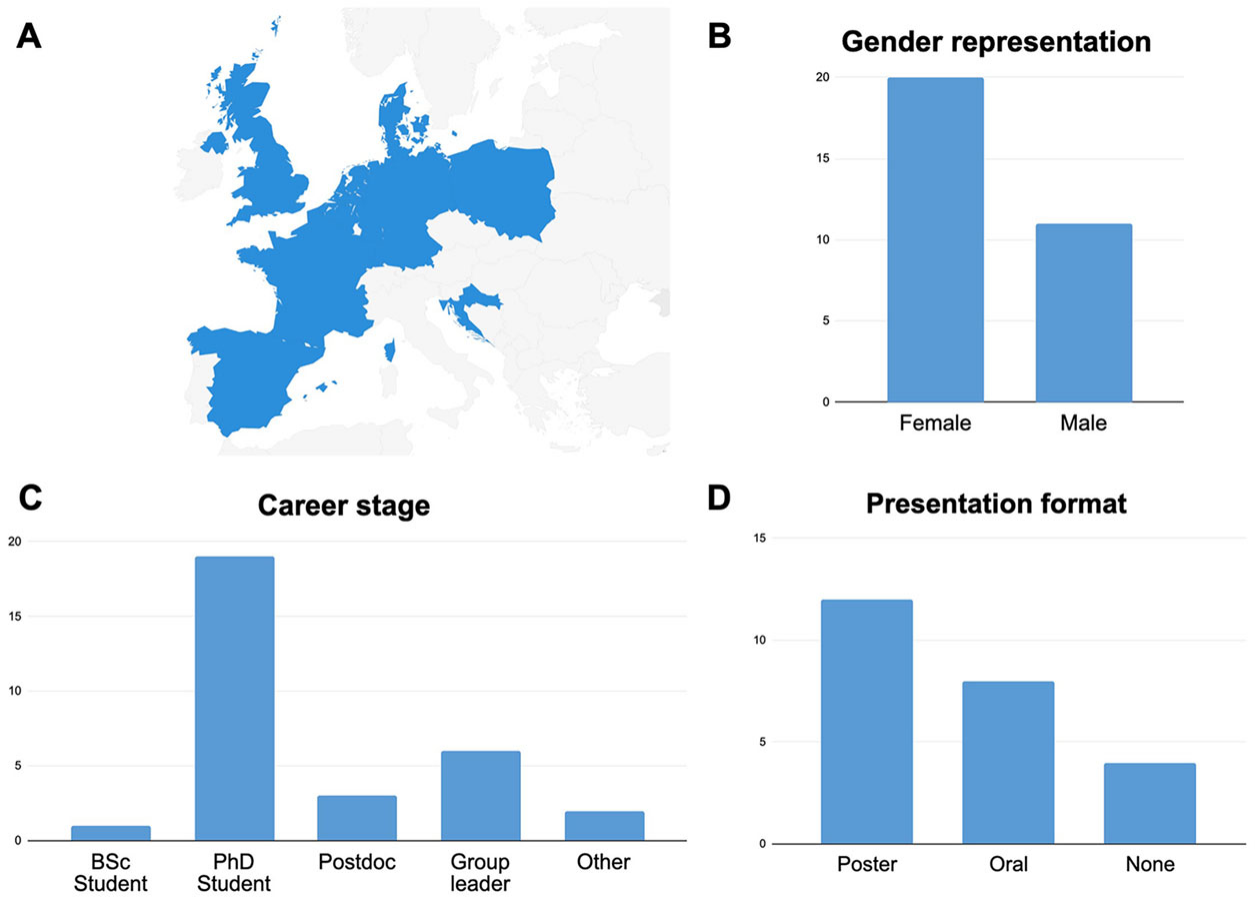
**Sociodemographic distribution of the attendees to the 14th NYRA meeting on 20-21 September 2022, in Norwich, UK.** Charts showcasing the (A) geographical distribution, (B) gender representation, (C) career stage, and (D) format of the selected presentations given by the delegates. Note that information about the invited speakers are included in all figures except in 1D.

The 14th NYRA meeting took place from 20–21 September 2022, with 31 participants from the UK, Croatia, Spain, Denmark, Germany, the Netherlands, France, Poland, and Belgium (Fig. 1A). About 65% of attendees were women (Fig. 1B). While over 60% of participants were PhD students, we welcomed researchers from various academic levels in the andrology field including undergraduate students, postdocs, group leaders, and industry representatives (Fig. 1C). The Scientific Program featured five plenary talks given by renowned invited speakers, who explored various aspects of andrology, such as (i) mutational processes operative in the germline, (ii) male germ cell development and ageing, (iii) the function of the germline epigenome, (iv) the role of the germ-stem cell niche in prepubertal testis development and function, and (v) germ cell development and fertility preservation. Delegates also presented their research work in the form of selected oral or poster presentations (Fig. 1D). We were honored to host two distinguished guests who led the workshops on transferable skills; Andrea Rippon, who introduced us to the amazing world of leadership skills, and Professor Simone Immler, who delivered a workshop on grant application writing with an interactive perspective.

## Scientific highlights of the 14th NYRA meeting

The first day of the 14th NYRA meeting was dedicated to the genetics of male infertility. Dr Raheleh Rahbari (Wellcome Sanger Institute, UK) opened the scientific part with an overview of the intricate mechanisms surrounding mutations in the male germline, specifically delving into germline aging and chemotherapeutics, and their impact on the predisposition of future generations to genetic diseases. Dr Rahbari's PhD student, Matthew Neville, continued the discussion by diving deeper into the characterization of human sperm mutations through innovative NanoSeq sequencing technology. His captivating lecture earned Matthew the NYRA award for the Best Selected Oral Presentation. The second plenary session was held by Dr Antoine Molaro (Genetic, Reproduction and Development, GReD, Institute, France) who shared his findings on the investigation of the germline epigenome. He showed results of the nucleosome destabilization activity of the short histone H2A variant, H2A.B, which evolved specifically for the testis, and that this variant can lead to oncogenesis when expressed in an inappropriate context. Dr Sandra Laurentino (Center of Reproductive Medicine and Andrology, University of Münster, Germany) focused on the impact of aging on spermatogenesis. She highlighted that while healthy aging does not negatively affect testicular function in terms of hormone production and sperm parameters, aging sperm is characterized by increased DNA fragmentation rates, changes in DNA methylation patterns, and longer telomeres. Professor Rod Mitchell (MRC Centre for Reproductive Health, University of Edinburgh, UK) closed the first day with a presentation on the different fertility preservation options available to children undergoing gonadotoxic cancer treatments who are not yet able to store a sperm sample. Furthermore, he discussed the benefits of a preventive, non-invasive approach to protect germ cells and testicular function during gonadotoxic exposure, based on the use of pharmaceuticals and their validation *in vitro*. The first day also featured engaging selected oral presentations from Dr Manon Oud (Donders Institute for Brain, Cognition and Behaviour, Radboudumc, the Netherlands), Dr Avinash Gaikwad (Institute of Reproductive Genetics, University of Münster, Germany), Dr Alberto de la Iglesia (Institute Cochin, University of Paris, France), Alice Godden (School of Biological Science, University of East Anglia, UK), and Afsaneh Khoskerdar (University of Nottingham, UK), who further explored the theme of (epi)genetics in male infertility.

Day two was centered on the biology of spermatogenesis with the fifth plenary session by Dr Kristian Almstrup (Department of Growth and Reproduction, University Hospital of Copenhagen, Denmark) on the importance of PIWI-interacting RNAs (piRNAs) for proper human spermatogenesis. The piRNA pathway regulates transposable elements in both fetal and adult testis, and gene expression in the adult testis. By doing so, piRNAs help ensure germline genome stability and proper functioning of the testis. The scientific program concluded with the selected oral presentations of Shruti Kane (School of Applied Sciences, University of Abertay Dundee, UK), whose talk was focused on the role of intracellular calcium signaling on sperm performance and Guillaume Richer (Biology of the testis lab, Vrije Universiteit Brussel, Belgium), who showcased the latest developments in testicular organoid formation.

**Fig. 2.**
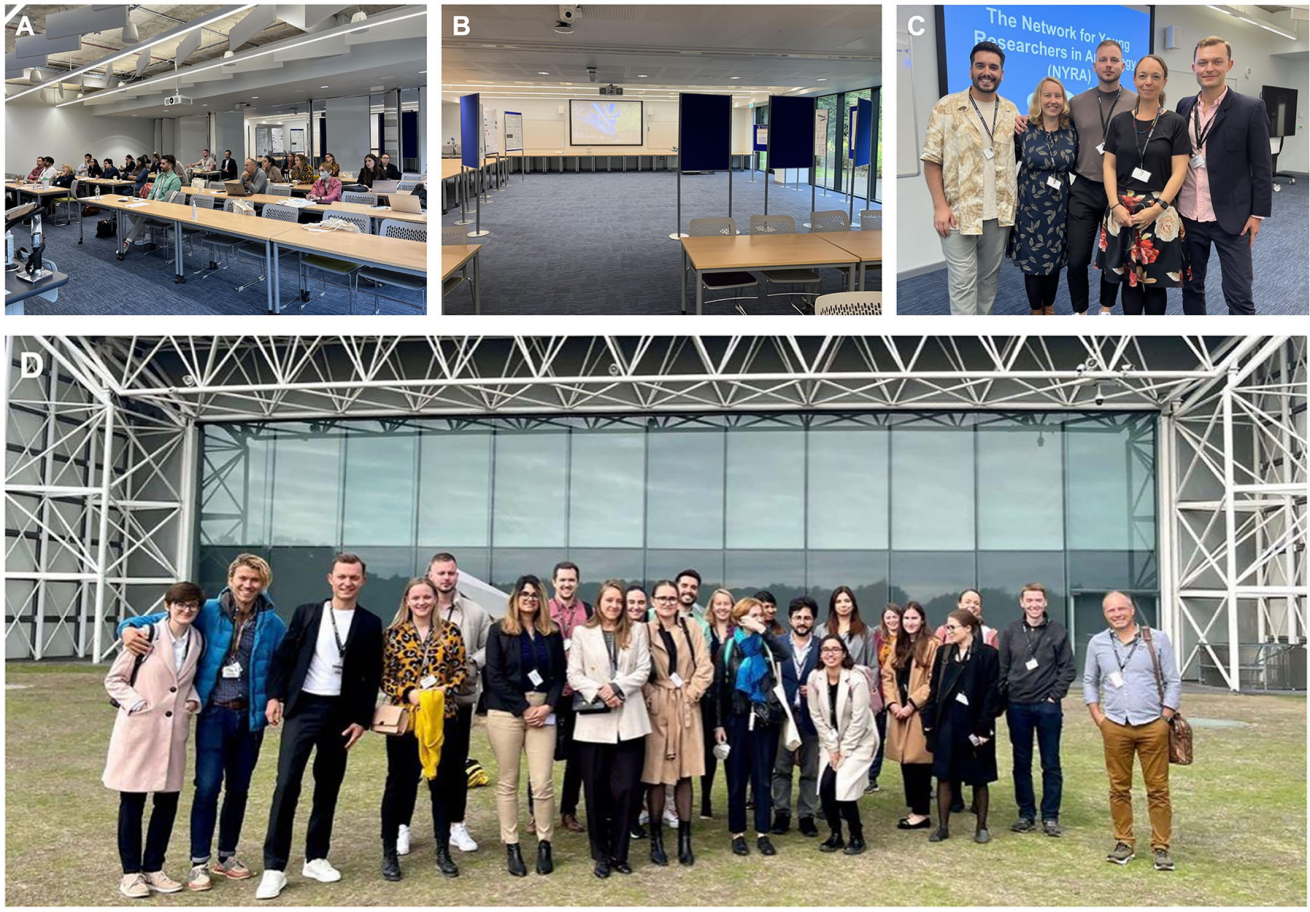
**Pictures of the 14th NYRA meeting.** (A,B) The conference room spaces for plenary and poster sessions, respectively. (C) Members of the NYRA Board attending the meeting: from right to left, Alberto de la Iglesia, Manon Oud, Guillaume Richer, Dorte Egeberg, and Daniel Marcu. (D) Attendees to the 14th NYRA Meeting in front of the Sainsbury Centre, one of the most prominent university art galleries in Britain, and a major national center for the study and presentation of visual art.

A total of five poster sessions were also held between plenary sessions, allowing attendees to delve deeper into the research and ideas presented. Amid the display of advances in male reproductive health research, Sylwia Lustofin (Department of Endocrinology, Jagiellonian University in Cracow, Poland) stood out as the deserving recipient of the Best Selected Poster Presentation award, acknowledging her study on the complex relationship between follicle stimulating hormone and notch signaling and their role in controlling Sertoli cell function. Overall, the high level of interaction helped to foster a relaxed and collaborative atmosphere among the attendees, including the renowned invited speakers (Fig. 2).

## Career development sessions

The number of students applying for PhD programs is increasing, but over half of them end up in careers outside academia, and fewer than 1% become full professors. Early-career researchers often struggle to identify and present the transferability of their academic skills to non-academic environments. The NYRA board, composed of members from varied professional backgrounds, understands the existing gap in information and guidance regarding developing skills for careers in and outside academia. To this end, the board of NYRA organizes workshops during their annual meetings featuring successful leaders in various areas to share their experience and provide practical training on ‘soft skills’ that can be applied to a wide range of jobs and industries. The 14th NYRA meeting delivered two interactive workshops that covered key skills necessary to become a successful researcher.

Andrea Rippon (Stronger Relationships limited, UK) presented the first workshop, entitled ‘Aspiring Leaders: Leadership is an Inside Job’. With over 20 years of coaching experience, Andrea has helped the NYRA participants build healthy mindsets in order to succeed in their personal and professional relationships. The workshop was primarily focused on equipping aspiring leaders with tools to lead themselves and inspire others. In small group discussions, the participants identified their own values, explored how to regulate their emotions, discovered new habits and self-motivation techniques to make better personal and professional decisions. Running the session in small groups allowed for a learner-centric approach, enabling each participant to explore their individual strengths within their respective group and receive tailored feedback. A major focus of the exercises was self-reflection and strategic implementation of leadership theories and concepts into everyday work life. According to Rippon, ‘although PhD students do not yet lead other people, self-leadership is essential for a successful PhD’. The participants appreciated that the leadership skills acquired during the workshop can be applied outside the academic setting.

Professor Simone Immler (School of Biological Science, University of East Anglia, UK) addressed how to handle the process of grant writing during the second workshop, entitled ‘The ever-shifting maze of careers in science’. With the increasing number of PhDs graduating and the scarcity of academic positions, young researchers often must secure funding to support their scientific endeavors. In fact, universities and research institutes often evaluate candidates based on their ability to win funding alongside their publication record. Understanding the art of successful fellowships and grant writing is therefore key for those aspiring to develop and lead their own scientific group. With an impressive record of attracting scientific funding, Professor Immler shared her personal and professional journey into academic research and provided key advice on being successful at obtaining fellowships and scientific grants. She started by sharing the funding successes and failures that she has experienced in her career and discussed the lessons she learnt from rejected grants. She argued that ‘rejections are often needed to learn how to navigate the ever-shifting maze in science’. A major part of the workshop was focused on understanding how to stand out in an application. In small groups, the participants had the opportunity to creatively develop research projects based on their shared interests and pitch them to a panel composed of the plenary speakers. Not only was this a useful exercise that allowed young scientists to be critically evaluated for their presentation skills, but they also developed a strong understanding of the grant and fellowship application process and interview format. Overall, the exercise encouraged active participation among the participants, which will be useful for potential future collaborations.

Professional and personal development workshops have become an integral part of the NYRA meetings. The resulting lessons and practical advice gained from these sessions can often be career defining. The NYRA board is committed to creating workshops on transferable skills to further expand the existing conference format shared by many meetings. We see this as an opportunity to turn young researchers into future leaders in both academic and industrial settings.

## NYRA Young Researcher Award 2022

**Fig. 3.**
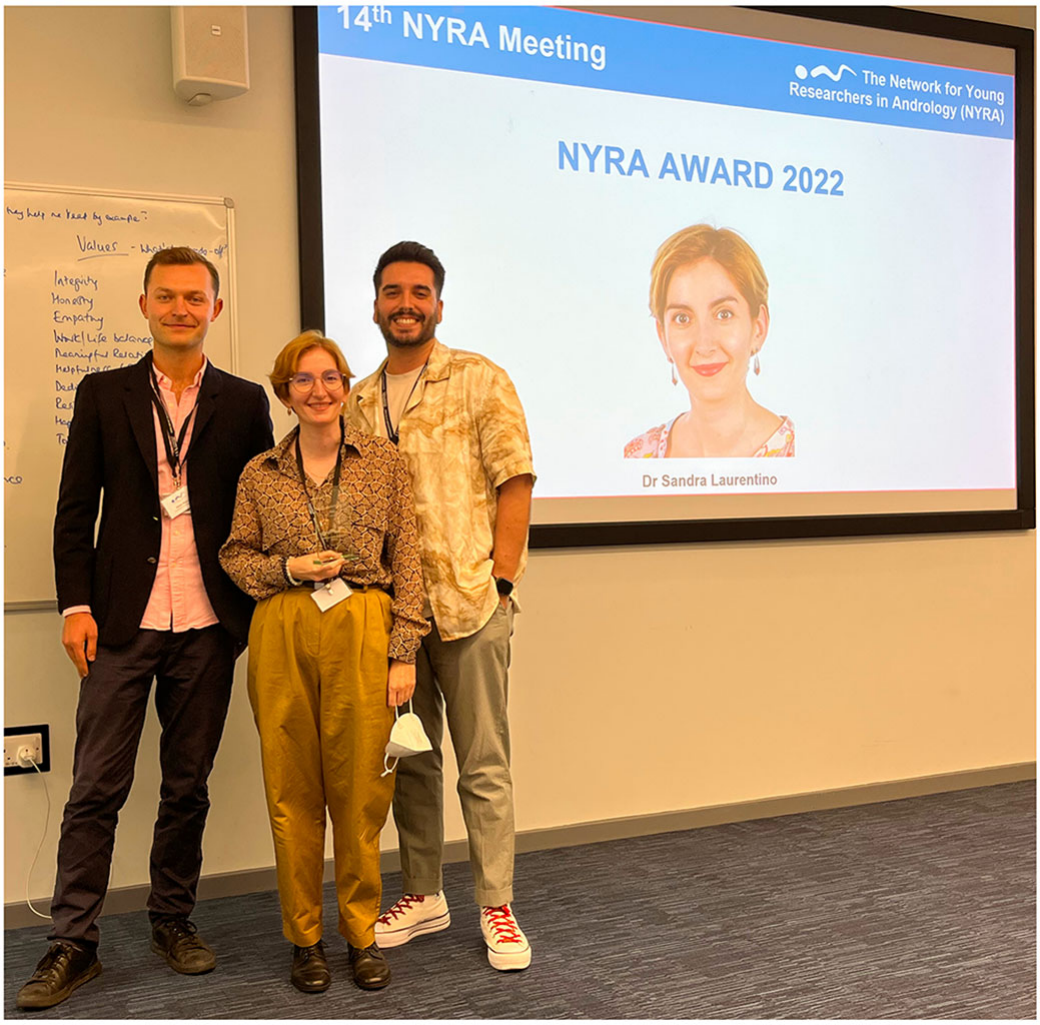
**Recognition to Dr Sandra Laurentino as the 2022 NYRA Young Researcher Award winner during the closing ceremony**. From left to right, Mr Daniel Marcu (14th NYRA meeting main organizer), Dr Sandra Laurentino, and Alberto de la Iglesia (NYRA President).

The NYRA Young Researcher Award is the highest recognition of our society, awarded biennially during our independent meetings in recognition of emerging group leaders who have demonstrated scientific independence, outstanding contribution to andrology, and mentorship of fellow young researchers. During the 14th NYRA meeting, Dr Sandra Laurentino impressed the NYRA board with her outstanding career and work on testicular gene regulation throughout male life. For her groundbreaking work, she was awarded with the Young Researcher Award 2022. Dr Laurentino is currently serving as the Principal Investigator at the Centrum für Reproduktionsmedizin und Andrologie at the University of Münster (Münster, Germany) (Fig. 3). In addition to deciphering transcriptomic and epigenetic changes associated with spermatogenic failure and human germline aging, Dr Laurentino has made significant contributions to the field of andrology, particularly in the areas of sex steroid receptors, epigenetics and novel tools to study Klinefelter Syndrome (46,XXY) testes.

## Sustainability

Following 2 years of virtual and postponed meetings due to COVID-19, we aimed to foster interactions between young researchers by encouraging participants to attend on site, while enabling virtual participation for those unable to attend. However, we also aimed to minimize the environmental footprint of the meeting by implementing sustainable practices throughout the event. One of these practices was the elimination of printed abstract books and encouraging attendees to consider whether they needed to print the electronically available program. Moreover, participants were encouraged to take public transportation when traveling to and from the meeting venue with approximatively half of the participants being local, reinforcing this sustainable practice. Of the invited speakers, one attended online, two traveled by train, and only two had to fly to the UK for the meeting. The workshop speakers were local, further reducing the transportation-related carbon footprint.

Attendees were encouraged to share accommodations in order to minimize energy usage which, together with all events, were held on the eco-friendly UEA campus, avoiding extra use of transportation. This way, it was possible to walk between the conference room and other locations on the campus. Meals were prepared and served at the campus canteen, which provided a range of vegetarian options, selected by more than half of the attendees. Recycle bins were available in the canteen and all over the campus, and packed lunches were served in recyclable packages to reduce waste. Overall, the 14th NYRA meeting successfully achieved its scientific and networking goals while also minimizing its environmental impact.
